# Congo Red as a Supramolecular Carrier System for Doxorubicin: An Approach to Understanding the Mechanism of Action

**DOI:** 10.3390/ijms23168935

**Published:** 2022-08-11

**Authors:** Klaudia Kwiecińska, Anna Stachowicz-Kuśnierz, Beata Korchowiec, Maciej Roman, Wojciech M. Kwiatek, Anna Jagusiak, Irena Roterman, Jacek Korchowiec

**Affiliations:** 1 Faculty of Chemistry, Jagiellonian University, 30-387 Krakow, Poland; 2 Institute of Nuclear Physics, Polish Academy of Sciences, 31-342 Krakow, Poland; 3 Faculty of Medicine, Medical College, Jagiellonian University, 31-034 Krakow, Poland; 4 Department of Bioinformatics and Telemedicine, Faculty of Medicine, Medical College, Jagiellonian University, 30-688 Krakow, Poland

**Keywords:** doxorubicin, Langmuir films, drug–membrane interactions, Raman spectroscopy, molecular dynamics simulations, drug carrier

## Abstract

The uptake and distribution of doxorubicin in the MCF7 line of breast-cancer cells were monitored by Raman measurements. It was demonstrated that bioavailability of doxorubicin can be significantly enhanced by applying Congo red. To understand the mechanism of doxorubicin delivery by Congo red supramolecular carriers, additional monolayer measurements and molecular dynamics simulations on model membranes were undertaken. Acting as molecular scissors, Congo red particles cut doxorubicin aggregates and incorporated them into small-sized Congo red clusters. The mixed doxorubicin/Congo red clusters were adsorbed to the hydrophilic part of the model membrane. Such behavior promoted transfer through the membrane.

## 1. Introduction

Doxorubicin (DOX) [1] is a drug commonly used in chemotherapy [2,3]. It was first approved for use almost fifty years ago. It belongs to the class of anthracycline drugs and is genotoxic to neoplastic cells [4,5]. DOX is used to treat many types of cancer including breast, leukemia, lymphoma, liver, lung, ovary, stomach, and thyroid. However, this versatility comes at a price—undesirable side effects. The most common of these include hair loss, vomiting, and allergic reactions. One should also mention cardiotoxicity leading to serious heart damage [6] and a drastic decrease in the number of myeloid cells [7]. The pharmacological activity of DOX is related to the presence of conjugated aromatic rings. The planar aromatic part of the molecule intercalates the DNA strand [8] and interacts with base pairs and thus disrupts the metabolism of highly replicable cancer cells. It blocks cell division and consequently leads to cell death. This effect is connected with the inhibition of topoisomerase enzyme [9]. Unfortunately, the same mechanism of action applies to healthy cells, which results in different side effects.

Strategies for avoiding undesirable side effects of doxorubicin [10] are associated either with design of a new generation of drugs or with reduction of the drug’s toxicity. The latter can be achieved by increasing its release at the target site. Targeted therapy is the subject of many studies—designing immunologically effective, specific drug-delivery carriers to tumor cells is of key importance in modern pharmacology [11]. The carrier should sustain drug release at the target site in a controlled manner and over a long time. In this way, the amount of drug reaching healthy cells diminishes and thus undesirable side effects are reduced. There have been many studies on the design of drug carriers [12,13,14,15,16], including polymeric carriers, inorganic nanoparticles, solid nanoparticles, polymeric hydrogels, macromolecular scaffolds, liposomes, and micelles.

A promising class of potential drug carriers, showing therapeutic effects, are self-assembling supramolecular structures [17,18,19]. An example of a species forming such self-organizing structures is Congo red (CR) [20]. CR is an anionic diazo dye with a planar, aromatic structure. In aqueous solution it forms ribbon-like supramolecular structures via stacking mechanism—CR units are stabilized by π–π interactions between the aromatic rings. Self-assembling CR structures have the ability to selectively interact with immune complexes. Binding to proteins [21,22], e.g., albumin, creates conditions for targeted immunotherapy. In addition, they can intercalate many drugs, including DOX, which cannot directly bind to albumin. Thanks to its unique binding properties, CR can be applied to detect fibril proteins enriched in a β-sheet conformation [21]. The increase of such conformers in proteins is a symptom of neurodegenerative pathologies (Alzheimer’s, Creutzfeldt–Jacob, Huntington’s, and Parkinson’s diseases). CR was also used as a DOX-accompanying molecule to enhance its release from hydrogels [23].

The aim of this study was to test the ability of CR to enhance DOX delivery to cancer cells. Raman spectroscopy, a sensitive and non-invasive imaging technique, was applied to study drug uptake and distribution in MCF7 breast-cancer cells. In vitro measurements were supplemented by monolayer experiments and molecular dynamics simulations. It was demonstrated that CR molecules fragment DOX oligomers and that DOX molecules intercalate CR clusters. This in turn increases adsorption to the monolayer and may favor the transfer through biological membranes.

## 2. Results and Discussion

### 2.1. Raman Spectroscopy

**Figure 1:**
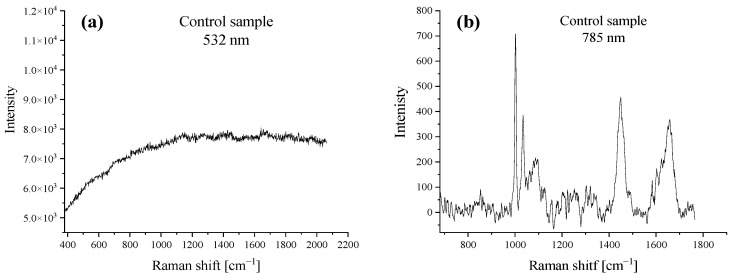
The spectrum of an MCF7 cell (control sample) recorded (**a**) with a 532 nm laser (dominated by fluorescence-DOX detection marker) and (**b**) with a 785 nm laser.

Figure 1a,b shows the Raman spectra of an MCF7 cell (control sample) for the 532 and 785 nm laser lines, respectively. The control sample was a breast-cancer cell not stimulated with the tested systems (CR, DOX, or DOX/CR). The spectrum in Figure 1a illustrates the natural fluorescence of a control cancer cell. The flat course of the spectrum indicates the absence of DOX in the cancer cell.

The spectrum shown in Figure 1b presents the cell specific bands e.g., 1000 cm^−1^ (phenylalanine), 1450 cm^−1^ (proteins and lipids), and 1660 cm^−1^ (mainly proteins). Measurements on the 785 nm laser line were carried out for each test cell to confirm the Raman signal quality of the cancer cell measured. The presence of the above-mentioned bands confirmed the correctness of the experiment.

**Figure 2:**
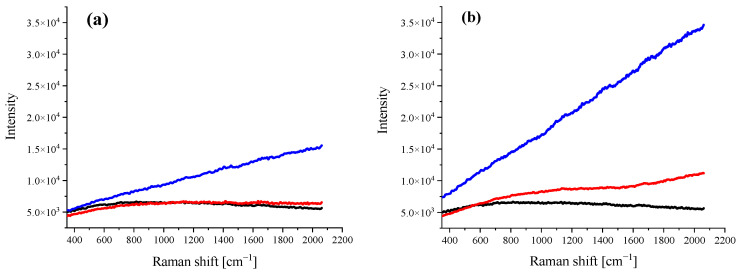
Comparison of Raman spectra recorded for a control (untreated) cell (black line), a cell treated with DOX (red line), and a cell treated with DOX/CR (blue line). Stimulation time: 24 h. DOX concentration: (**a**) 1 nM and (**b**) 100 nM.

Figure 2 shows Raman spectra obtained with a 532 nm laser line for a control cell (black), a DOX-treated cell (red), and a DOX/CR-treated cell (blue) for concentrations of 1 and 100 nM and stimulation time of 24 h. For a concentration of 1 nM (panel a) it was observed that, in the absence of CR carrier, DOX does not enter the cell, while in the mixed DOX/CR system a spectrum dominated by fluorescence was obtained. For the higher concentration of 100 nM (panel b), a slight increase in spectrum course it was noticed, indicating that DOX gently penetrates the cell. However, the spectrum for the DOX/CR system is much clearer and indicates a much greater accumulation of the drug in the cancer cell.

The results suggest that DOX without the carrier does not penetrate the cell (or it penetrates very little at higher concentrations and longer stimulation times). When CR is used as a carrier, a spectrum dominated by DOX fluorescence was obtained. This phenomenon was already observed at the lowest applied concentration of the drug, 1 nM. The obtained results indicate that the use of a drug carrier in cancer therapy may contribute to the reduction of the effective dose of the drug and, consequently, may reduce the side effects associated with chemotherapy.

**Figure 3:**
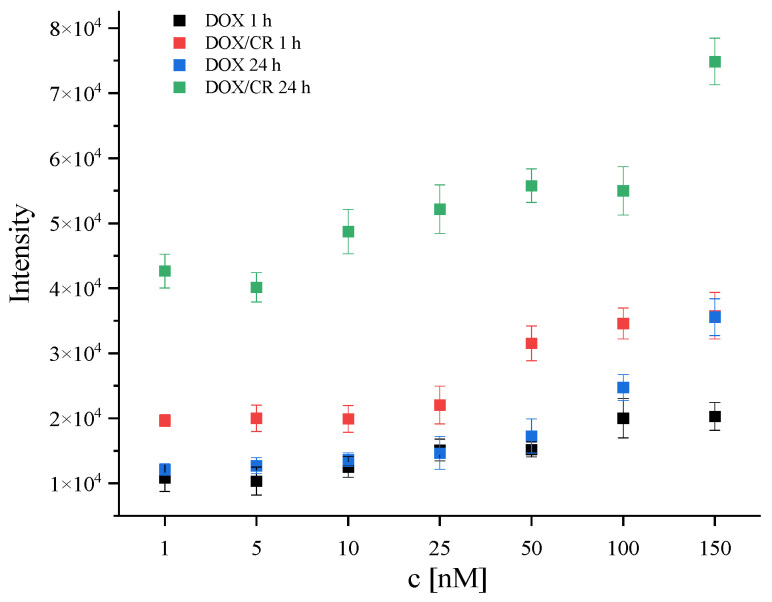
Accumulation of DOX in MCF7 cells treated with DOX or DOX/CR complexes as a function of DOX concentration. Accumulation was quantified as Raman intensity at 2000 cm^−^^1^ (see main text for details). Spectra were obtained with the excitation wavelength of 532 nm. Results for cells treated with DOX for 1 h (black) and 24 h (blue) are compared with those of cells treated with DOX/CR complexes for 1 h (red) and 24 h (green).

In order to quantify the observed effect, for each sample containing DOX (DOX and DOX/CR in all tested concentrations) Raman intensity at 2000 cm^−1^ was read from each spectrum (excitation wavelength of 532 nm) and arithmetic means and standard deviations were calculated. The results are presented in Figure 3. It can be seen that the use of CR as a carrier for DOX transport increases the penetration of the drug when compared to using DOX without the carrier. A positive effect was already observed at the DOX concentration of 1 nM (lowest applied) and with a shorter stimulation time (1 h). Accumulation of DOX in tumor cells threated with DOX/CR complexes at the lowest concentration tested (1 nM, 1 h) was comparable to the accumulation in the cells treated with DOX alone at a stimulation time of 1 h and concentration 100 times higher (100 nM). These results also indicate stimulation time as an important issue. Longer incubation time between the drug/carrier system and tumor cells allows the dose of the drug to be reduced, which may contribute to reduction of the side effects of chemotherapy.

**Figure 4:**
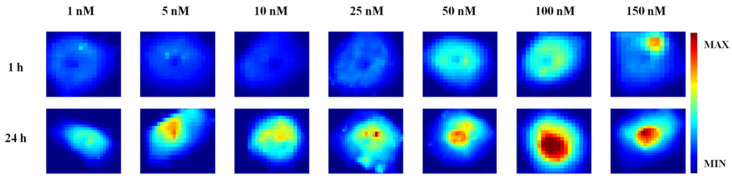
Raman maps showing the accumulation of DOX in MCF7 cells treated with DOX/CR complexes for varying DOX concentration and stimulation time.

Figure 4 shows the distribution of DOX content over the entire cell area for all tested concentrations (c = 1, 5, 10, 25, 50, 100, and 150 nM) and stimulation times (1 and 24 h) based on Raman intensity at 2000 cm^−1^. The presented maps suggest that DOX gradually enters the cell with increasing concentration and stimulation time and it tends to accumulate in the area of the cell nucleus (red-brown areas in the maps). The presented results indicate that the use of longer stimulation time is justified and contributes to greater effectiveness of the drug penetration into neoplastic cells.

### 2.2. Monolayer Experiments

#### 2.2.1. Compression Isotherms

To better understand the mechanism of DOX entry into MCF7 cells, the Langmuir film technique was used. The interaction of active substances, including drugs or their carriers, with lipids present in the cancer-cell membrane may be crucial in understanding how cancer cells are destroyed during treatment. Phosphatidylcholine is the most common component of the lipid cell membrane [24,25,26,27]. In our work, DPPC was chosen as the model membrane lipid. Since lipids are amphiphilic, water-insoluble compounds, monomolecular Langmuir films were applied [28,29]. The DPPC monolayer was formed on buffered saline, as well as saline containing DOX, CR, or DOX/CR. This method allows the determination of membrane–drug interactions in the presence or absence of potential drug carrier molecules [30,31].

**Figure 5:**
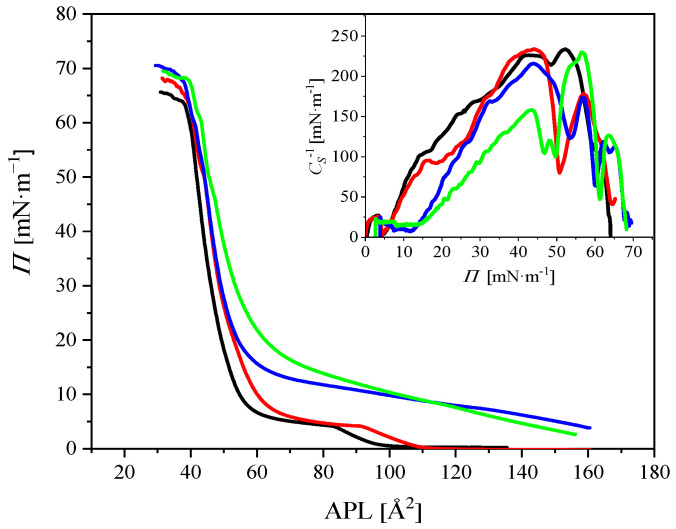
Compression isotherms of DPPC monolayer spread on PBS (black line), DOX (red line), CR (green line), and DOX/CR (blue line) subphase. DOX concentration was 1 μM; *T* = 20 °C.

The surface pressure isotherms obtained upon compression of the DPPC monolayer at 20 °C are shown in Figure 5. The inset in the figure shows the plots of compressibility modulus vs. surface pressure (*C*_S_^1^–*Π*). The DPPC monolayer was spread on PBS solution (black line) as well as PBS with DOX (c = 1 μM, red line), CR (c = 2 μM, green line), or DOX/CR (1:1 *v/v* ratio, blue line). The results obtained for higher DOX and CR concentrations are included in the Supplementary Materials (Figure S1).

The isotherm obtained on the DOX solution differed from that on the PBS. Indeed, the isotherm corresponding to the monolayer spread on the subphase containing DOX was shifted to higher molecular areas; the shift was greater with increasing DOX concentration (Figure S1). Moreover, the slope of the isotherm remained unchanged, showing similar phase characteristics of the monolayer with a clearly marked phase transition (Figure 5, the inset). The liquid expanded–liquid condensed (LE–LC) phase transition appeared at the same surface pressure (*Π* = 4.5 mN∙m^−1^) compared to that of the monolayer spread on PBS. However, for a DOX concentration of 5 and 10 μM, the LE–LC phase transition shifted to 6.3 and 7.2 mN∙m^−1^, respectively, showing the greater fluidizing effect on the DPPC monolayer.

The molecular area effect was also observed when CR was present in the subphase. In this case, the increase in molecular area was more significant compared to the DPPC/DOX system. The DPPC monolayer formed on the CR solution was more fluid, as indicated by the lower values of the compressibility modulus and did not show the LE–LC phase transition plateau characteristic of the DPPC isotherm. The above results may indicate that CR penetrated into the monolayer from the subphase, significantly changing the molecular orientation and order of the monolayer, while DOX would adsorb to the monolayer and interact with the polar heads of DPPC.

When both DOX and CR were present in a subphase, the resulting isotherm was intermediate between the two isotherms formed on DOX or CR subphase; the same relationship applied to the compressibility modulus. In the range of high molecular area, the isotherm more closely corresponded to the isotherm spread on the CR subphase, while in the low molecular areas it was in line with the isotherm on the DOX subphase. Interestingly, for the highest available areas, the isotherm achieved even higher values of surface pressure (and same monolayer was more fluid) than that on the CR subphase. This effect was more important at higher DOX concentrations (Figure S1a,b), which may indicate that both substances present in the subphase were penetrating into the DPPC film. Moreover, the surface pressure at the collapse point of the monolayer was highest when DOX/CR was present in the subphase, which confirms the highest stability of the monolayer.

#### 2.2.2. Adsorption Kinetics

**Figure 6:**
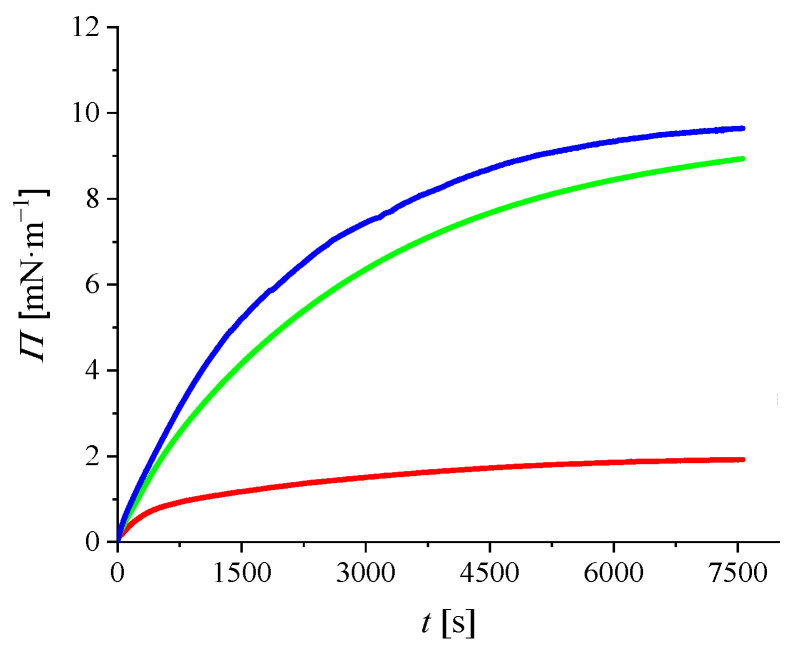
Adsorption kinetics of DOX (red line), CR (green line), and DOX/CR (blue line) to the DPPC monolayer. Initial surface pressure, *Π*_0_ = 28 mN∙m^−1^, was normalized to 0.

Figure 6 shows the adsorption kinetics of DOX, CR, and DOX/CR to the DPPC film, determined as changes in surface pressure (Δ*Π*) as a function of time. The isotherms were measured for initial surface pressure *Π*_0_ = 28 mN∙m^−1^, which corresponds to the lateral pressure in biological membranes [32]. In order to facilitate the observation of the effect of DOX and CR on the model membrane, the *Π*_0_ value was normalized to zero.

A slight increase in surface pressure could be observed with DOX. This can be attributed to the adsorption of DOX on the DPPC monolayer. In the case of CR or DOX/CR in the subphase a sharp increase in surface pressure was observed; the effect was larger with DOX/CR. These results show that DOX penetrates from the subphase into the lipid monolayer in the presence of CR and is involved in modifying the molecular organization of the DPPC.

Based on the monolayer experiments, it can be assumed that the presence of CR allows DOX to penetrate into the DPPC monolayer. These results are consistent with those obtained from the Raman studies.

### 2.3. Molecular Dynamics Simulations

Interpretation of experimental measurements requires more detailed research. Here, molecular dynamics simulations were used to explore the transport capabilities of DOX molecules across a model cell membrane. Theoretical models closely related to experimental data allow better understanding of the interactions between molecules at atomic resolution [33,34,35,36].

#### 2.3.1. Potential of Mean Force Calculations

**Figure 7:**
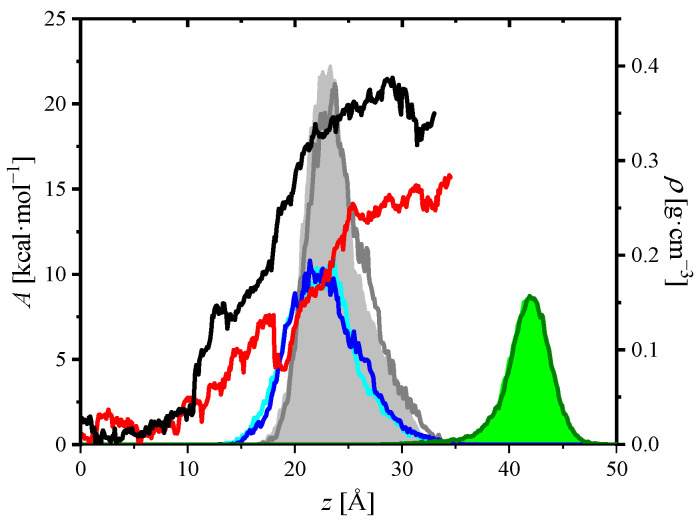
Free energy profiles ΔA=ΔA(z) related to position of DOX (black line) and CR (red line) along the monolayer normal (*z*-axis) imposed on partial density plots $\rho_{X}=\rho_{X}\left(z\right)$. The color code for partial density plots is as follows: cyan line (N(CH_3_)_3_ in DOX/DPPC system), blue line (*X* = N(CH_3_)_3_ in CR/DPPC system), filled light grey area (*X* = PO_4_ in DOX/DPPC), grey line (*X* = PO_4_ in CR/DPPC), filled green area (chain terminal CH_3_ in DOX/DPPC), and olive line (chain terminal CH_3_ in CR/DPPC).

In Figure 7 free energy changes accompanying transfer of DOX or CR molecules across DPPC monolayer are plotted. The *z*-coordinate of the center of mass of DOX or CR molecule was chosen as a reaction coordinate (RC). Calculations were performed using potential of mean force technique. The black and red curves correspond to DOX and CR molecules, respectively. To relate these curves to the DPPC monolayer, the figure includes partial density plots of selected groups of DPPC along the *z*-axis. The hydrophilic N(CH_3_)_3_, PO_4_ groups and hydrophobic terminal CH_3_ groups were taken into account. The light gray and green filled areas correspond to the PO_4_ and CH_3_ units in the DOX/DPPC system, respectively. The equivalent density profiles in the CR/DPPC system are marked with gray and olive lines, respectively. The choline groups are marked with cyan (DOX/DPPC) and blue (CR/DPPC) lines. The density profiles of equivalent groups overlap, small differences can be attributed to interactions with DOX or CR. Such a pattern was expected; except for the perturbing DOX and CR molecules, both systems had the same number of atoms and calculations were performed under the same physicochemical conditions (canonical ensemble). In the water bulk (z<10 Å), the free energy of DOX or CR fluctuated and changes did not exceed 2 kcal∙mole^−1^. As the molecules approached the monolayer, free energy increased. However, there was a clear minimum in free energy profile of CR/DPPC system located at *z* = 18–19 Å. A shift towards higher *z* values resulted in further increase in free energy. Roughly speaking, at the interface (10 Å<z<30 Å—hydrophilic area) the DOX/DPPC curve was about 5–10 kcal∙mole^−1^ above the CR/DPPC one. In conclusion, two general observations follow from ΔA=ΔA(z) plots: (*i*) the free energy cost of transfer through the membrane is lower for CR than for DOX and (*ii*) CR forms complexes with DPPC at the early step of RC.

**Figure 8:**
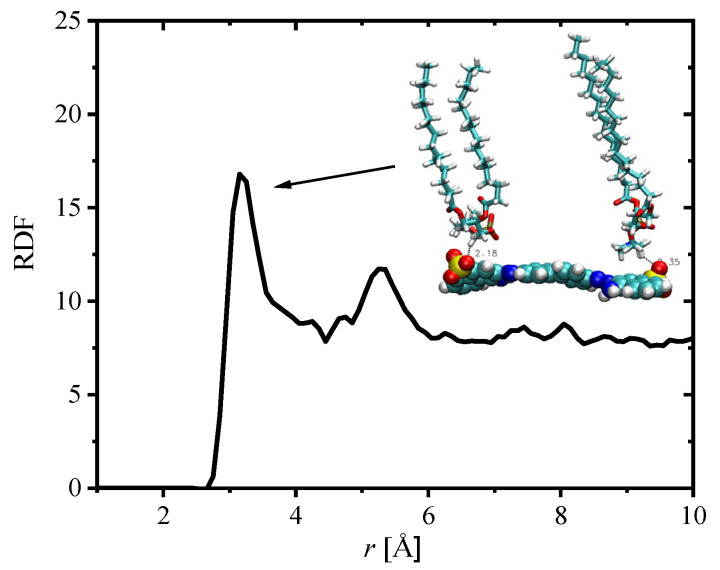
Radial distribution function representing the distribution of the carbon atoms of the N(CH_3_)_3_ choline group around the SO_3_ oxygen atoms of CR. The inserted drawing illustrates the corresponding binding mode.

Examination of the trajectories reveals that the interactions between SO_3_ groups of CR with choline hydrogen atoms are responsible for formation of CR–DPPC complexes. Such complexes were located below the monolayer, in the water subphase. The bonding pattern is confirmed by radial distribution function (RDF) between oxygen atoms from SO_3_ groups of CR and the choline carbon atoms from DPPC, shown in Figure 8.

A clear maximum can be seen in the RDF plot, showing that complexation between these groups occurred. The observed minima are rather shallow. The structure of an example CR–DPPC complex is shown in the inset to the figure. The probable driving force for formation of these complexes is electrostatic attraction between negatively charged SO_3_ and positively charged choline groups. Similar complexes were not observed in DOX/DPPC system.

#### 2.3.2. Unbiased MD Simulations

Both DOX and CR form oligomers in water. The same is true for a mixed DOX/CR system. It is illustrated in Figure S2 of the Supplementary Materials for symmetric monolayer models. Due to the system size, the calculations could not cover long time-scales. However, they are very instructive. Small clusters of different sizes are observed in the CR/DPPC model. On the other hand, DOX forms huge clusters in the subphase (Figure S2b). In the presence of CR, the DOX oligomer is broken into smaller clusters (Figure S2c). The CR, thanks to two flat, slightly twisted units, creates short ribbon-like structures. Unlike the CR, the flat DOX unit is too small to form a regular stack. Moreover, other non-planar DOX subunits promote the formation of irregular oligomers. CR extracts planar DOX units from the oligomer and intercalates them between the CR. Often, two planar DOX moieties are interposed between two CR molecules. DOX sugar residues cover the outer surface of the ribbons. A similar mechanism has been postulated to explain DOX interaction with DNA. In summary, it can be said that CR cuts the DOX oligomer and intercalates DOX molecules between the scissor’s blades.

**Figure 9:**
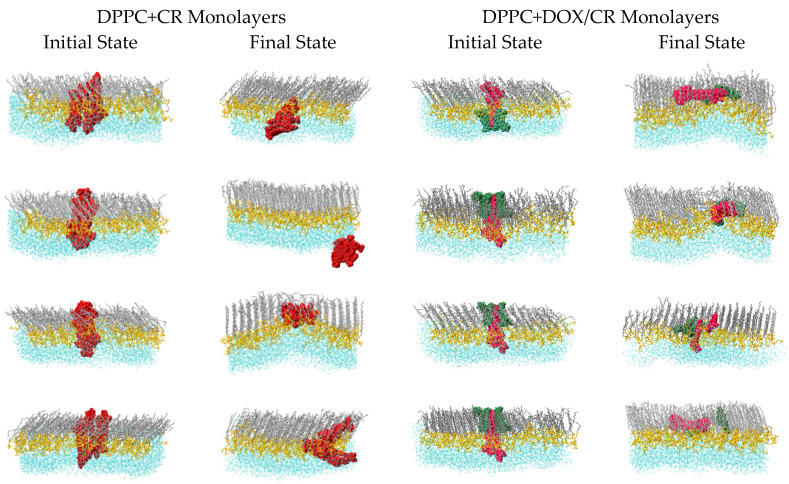
Initial configurations and final structures obtained in the AA–MD simulations of CR (**left**) and DOX/CR (**right**) clusters in DPPC monolayers. Simulations were performed at *p_z_* = 1 atm, *T* = 293 K, and *γ* = 40 mN∙m^−1^. Color code: DPPC chains–gray, DPPC heads–yellow, CR–red, DOX–green, and water–cyan.

As the next step of the modelling process, simulations of CR and DOX/CR clusters in DPPC monolayers were performed. Clusters were manually inserted into DPPC monolayers and their behavior was studied by means of MD simulations. Equilibration in the (N,V,T) ensemble was performed first. It was followed by simulations in the $\left(N,\gamma ,p_{n},T\right)$ ensemble with target γ= 40 mN∙m^−1^. Configurations of the initial and final state of the studied systems are shown in Figure 9.

In the case of CR/DPPC systems three scenarios have been observed for the fate of the CR cluster in DPPC monolayer. Firstly, in two systems (first two rows in the left-hand half of Figure 9), CR clusters were expelled from the hydrophobic part of the monolayer. Secondly, in one system (third row) the cluster remained in the monolayer and located on top of a fold formed by the monolayer. Finally, in one system (last row), the cluster was divided: two of the five CR molecules remained in the hydrophobic part of the monolayer, while three molecules were pushed out. On the other hand, in the DOX/CR/DPPC systems, all DOX/CR clusters remained in the monolayer. Two patterns were observed in these simulations. In two systems (first two rows in the right-hand half of Figure 9), the monolayer folded significantly, and the cluster was located on top of the fold. In the remaining two systems (third and fourth rows) the clusters were divided. In the first system one CR molecule detaches from the cluster. In the second, one DOX molecule detaches. However, in contrast to CR/DPPC systems, all of the fragments remained in the hydrophobic part of the monolayer. Detached molecules intercalate between the lipid chains and adopt tilt angle similar to that of the chains.

**Figure 10:**
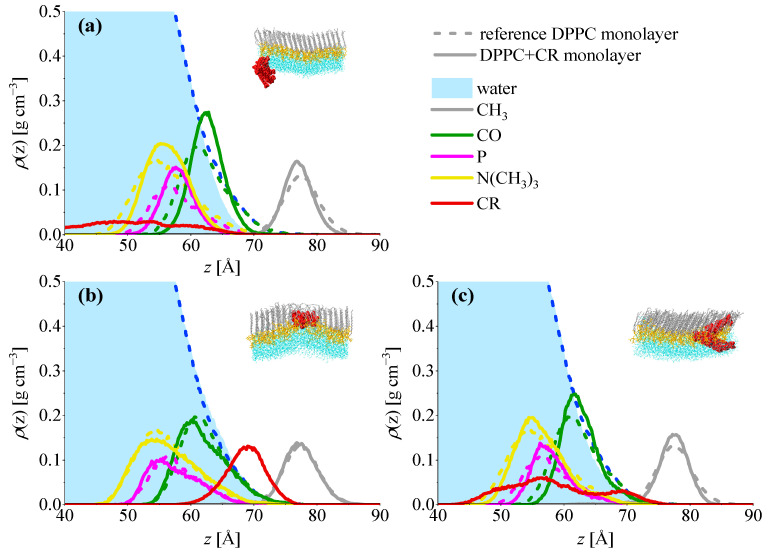
Partial density profiles along *z* axis in DPPC+CR monolayers compared with reference DPPC system. (**a**) Systems in which the CR cluster is expelled from the monolayer, (**b**) system in which the CR cluster is located on top of a monolayer fold, and (**c**) system in which the CR cluster is divided. Example structures of respective DPPC+CR systems are presented in the insets. Color code: DPPC tails–gray, DPPC heads–yellow, CR–red, and water–cyan.

The above observations are confirmed when partial density profiles along *z*-axis, ρ=ρ(z), are analyzed. CR/DPPC systems are presented in Figure 10. The plot in panel (a) was averaged over the two systems in which the CR cluster was expelled from the monolayer. It can be seen that the ρ(z) plot for the CR molecules (red line) overlaps with the plots for lipid headgroup components and extends far towards the subphase. Panel (b) shows the plots for the system where the CR cluster is located on top of the monolayer fold. In this case the ρ(z) plot for CR is located in the hydrophobic region and only partially overlaps with the plot of water. Finally, in the system where the cluster is divided (panel c), ρ(z) of CR is split and two maxima can be seen. One of them is located in the headgroup region, while the second is in the hydrophobic region.

**Figure 11:**
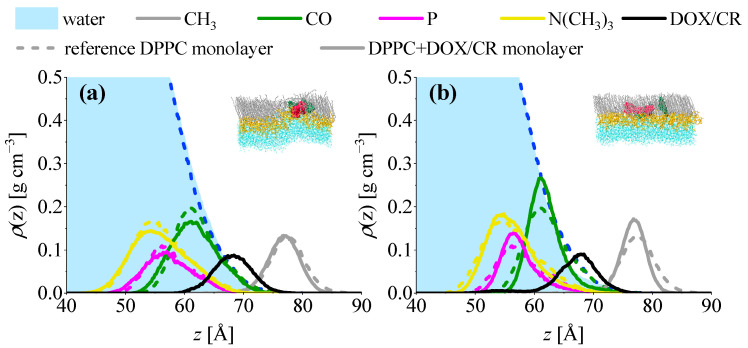
Partial density profiles along *z* axis in DPPC+DOX/CR monolayers compared with reference DPPC system. (**a**) Systems in which DOX/CR cluster is located on top of a monolayer fold and (**b**) systems in which DOX/CR cluster is divided. Example structures of respective DPPC+DOX/CR systems are presented in the insets. Color code: DPPC tails–gray, DPPC heads–yellow, CR–red, DOX–green, and water–cyan.

Partial-density plots for the DOX/CR/DPPC systems are presented in Figure 11. Panel (a) shows ρ(z) plots averaged over the two systems in which DOX/CR clusters are located on top of the monolayer fold. In panel (b) averaged plots for the systems with divided DOX/CR clusters are presented. In both cases, plots for the clusters (black lines) are located in the hydrophobic region of the monolayer. The difference between these two cases is manifested in the plots for headgroup components. In the first case, the monolayer is strongly folded, which is reflected in broad *ρ*(*z*) plots for choline (yellow lines), P atom (magenta), and CO groups (green). In the second case, less folding is observed, and the peaks for the headgroup components are sharper.

To conclude this part of the study, it can be stated that, at the selected surface tension of 40 mN∙m^−1^, CR clusters exhibit higher tendency to leave the hydrophobic part of the monolayer than the mixed DOX/CR aggregates. This observation can be explained in terms of the interaction between positively charged choline groups of the lipids and SO_3_ groups in CR molecule, identified in the previous section. This tendency may be used to rationalize the observations that, at high surface pressures, the experimental isotherms of DPPC+CR and DPPC+DOX/CR monolayers shown in Figure 5 converge to the isotherm of DPPC+DOX monolayer.

## 3. Materials and Methods

### 3.1. Materials

**Figure 12:**
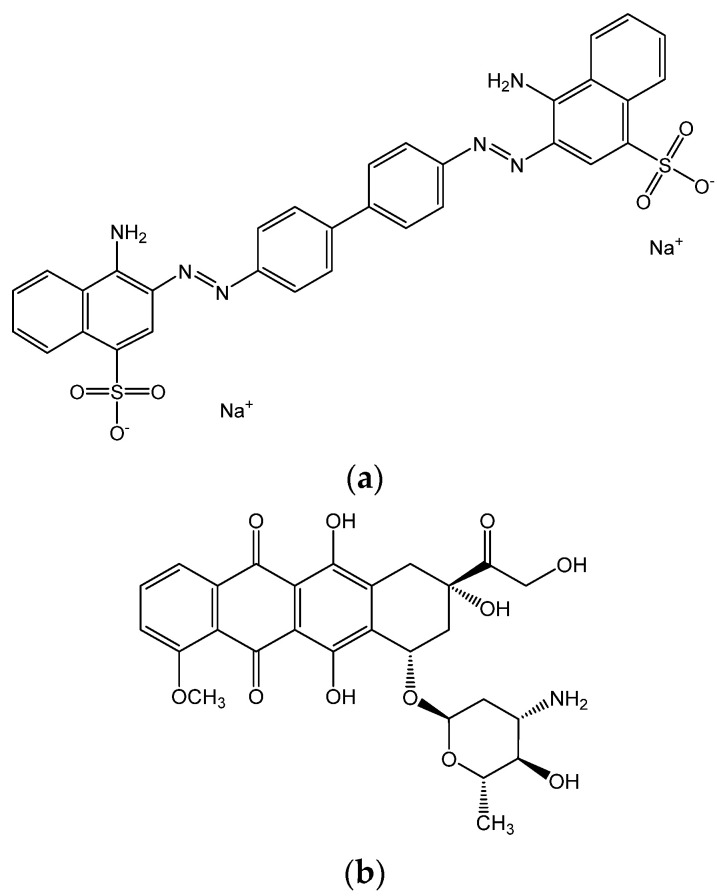
Chemical structure of (**a**) Congo red (CR) and (**b**) doxorubicin (DOX).

Doxorubicin hydrochloride (DOX, purity 97.9%) was purchased from Supelco. Congo red (CR, 96% pure), phosphate buffered saline (PBS), and chloroform (~99.9% pure) used in the preparation of phospholipid solution were from Sigma-Aldrich Chemie GmbH—Schnelldorf, Germany. Chemical structures of doxorubicin hydrochloride and Congo red are shown in Figure 12. 1,2-dipalmitoyl-*sn*-glycero-3-phosphocholine (DPPC) with a purity of >99% was obtained from Avanti Polar Lipids. Aqueous solution of PBS and PBS containing DOX (1, 5, and 10 µM), CR (2, 10, and 20 µM), or DOX/CR mixture (1:1 *v/v* ratio) were used as subphases. PBS buffer was prepared using ultrapure water (MilliQ, Millipore SAS, Molsheim, France) with a resistivity of 18 MΩ∙cm and surface tension of 72.8 mN∙m^−1^ at 20 °C.

The breast-cancer cell line (MCF7) was purchased from ATCC (American Type Culture Collection, Manassas, VA, USA). The cells were grown in DMEM culture medium (Dulbecco’s Modified Eagle Medium) containing 10% fetal bovine serum (FBS, EURx Sp. z o.o., Gdansk, Poland), insulin (0.01 mg∙mL^−1^), penicillin (100 U∙mL^−1^), and streptomycin (100 μg∙mL^−1^). Cultures were carried out at 37 °C with 5% CO_2_ content until 80% culturing area was covered. The passage was carried out using 0.25% trypsin. Then, the cells were seeded on culture plates containing CaF_2_ windows, changing the medium every 24 h. Cells were grown until 80% of the culture area was covered. After completion of the culture, the cells were stimulated with the appropriate amount of the tested systems (CR, DOX, and DOX/CR) with the appropriate concentrations of CR (80 μM), DOX (1, 5, 10, 25, 50, 100, and 150 nM) and DOX/CR (CR constant concentration 80 μM, DOX concentration increasing as above). Incubation of samples prepared in this way continued for 1 or 24 h. After incubation the cells were washed twice with the culture medium (2 mL), fixed with 2% glutaraldehyde in PBS (2 mL, 1 h), and washed twice with PBS (2 mL). Before the further analysis, the cells were stored in 3 mL of PBS at 4 °C.

### 3.2. Raman Spectroscopy

Raman measurements were performed using a Renishaw InVia Raman spectrometer equipped with an optical confocal microscope, air-cooled solid state lasers emitting at 532 and 785 nm, and CCD detector thermoelectrically cooled to −70 °C. An Olympus LUMPLFL N objective (60×, NA 1.0, water immersion) was applied for measurements in PBS. The power of the laser at the sample position was approximately 14 mW and 22 mW for the 532 nm and 785 nm laser line, respectively. The 532 nm laser line was used to detect DOX in cells (at this wavelength the spectrum is dominated by fluorescence which is a good marker for DOX detection). The excitation wavelength of 785 nm was used to confirm the correct measurement of the cell spectrum thru occurrence of the cell-specific bands e.g., 1000 cm^−1^ (phenylalanine), 1450 cm^−1^ (proteins and lipids), and 1660 cm^−1^ (mainly proteins) since the spectrum obtained using the 532 nm laser line is not characteristic. The 785 nm laser line was also used to record the Raman spectrum of CR. The spectrum showed three characteristic high-intensity bands (1157 cm^−1^, 1373 cm^−1^, and 1592 cm^−1^), which were considered as marker bands of the CR presence. Since no marker bands were observed in Raman spectra of the cells, no accumulation of CR in cells was proved. However, this does not mean that CR was not present in cells at all. Instead, it can be stated that the final concentration of CR in cells was below the detection limit of Raman measurements with the applied parameters. The CR spectrum was obtained for a solution with a concentration of 0.5 mg∙mL^−1^ (720 μM). CR concentration in DOX/CR complexes in cells was lower (80 µM). For each sample, Raman spectra of at least five cells were collected. For mixed the DOX/CR system, Raman mapping was also performed. The size of each map depended on the cell dimensions, i.e., the whole cell area was mapped with a step size of 1 μm. A sum of 1 scan with an integration time of 0.5 s was collected from each point. Raman spectra of DOX were measured as a 1 scan using 7 mW laser power and 1 s integration time, whereas spectra of cells and CR were collected as a sum of 5 scans with an integration time of 3 s using 14 mW laser power. All spectra were collected with a spectral resolution of ca. 1.5 cm^−1^. The spectrometer was calibrated using the Raman scattering line generated by an internal silicon plate.

### 3.3. Surface Pressure–Area Isotherms

The surface pressure (*Π*) measurement was carried out using a KSV 2000 Langmuir balance (KSV Instruments, Helsinki, Finland). A Teflon trough (58 × 15 × 1 cm) with two hydrophilic Delrin barriers providing a symmetric compression was used in all experiments. Surface pressure was measured with a platinum Wilhelmy plate. The apparatus was closed in a Plexiglas box and the temperature was kept constant at 20 °C. Before each measurement, all impurities were removed from the subphase surface by sweeping and suction. Monolayers were spread from chloroform solutions of accurate DPPC concentrations using a microsyringe (Hamilton Co., Reno, Nevada, USA). After the equilibration time of 20 min, the films were compressed at the rate of 5 mm∙min^−1^ by two symmetrically moving barriers. A PC computer and KSV software were used to control the experiments. Each compression isotherm was performed at least three times. The accuracy of the results was ±0.1 Å^2^ for area per lipid (APL) and ±0.01 mN∙m−^1^ for surface pressure.

The surface pressure–area isotherms allowed the compressibility modulus (*C_s_*^−1^ = –*A*(d*Π*/d*A*)*_T_*) [37,38,39] to be determined.

### 3.4. Adsorption Kinetics Measurement

The adsorption experiment was carried out in a glass vessel containing 20 mL of the subphase. Chloroform solution of DPPC was spread at the surface of the buffer solution using a Hamilton syringe (the solution was applied until the monolayer reached the desired surface pressure). After 20 min of monolayer equilibration, a small volume of concentrated DOX or CR solutions was injected into the subphase (under the DPPC monolayer) in amounts appropriate to achieve a subphase concentration of 1 or 2 µM for DOX or CR, respectively, and a 1:1 *v/v* ratio for the mixture. The subphase was slowly stirred with a magnetic stirrer for 30 s. The adsorption kinetics were recorded for over 2 h using a KSV Langmuir balance. All measurements were performed at 20 °C.

### 3.5. MD Simulations

A symmetric model of DPPC monolayers was used to investigate behavior of DOX and CR molecules in the model membrane environment. The model consisted of two monolayers spread on the opposite sides of water slab. DPPC monolayers were located in *xy* plane. Calculations were performed using periodic boundary conditions. The dimension of the simulation box in *z*-direction, normal to the monolayer, was enlarged with respect to the system size. This technical treatment introduces the air-water interface. The empty space between repeating system replicas corresponds to vacuum and replaces the air. The *z*-size of the vacuum slab was enlarged to minimize interactions among translational replicas. The same condition was imposed on the size of the water slab, i.e., it was big enough to minimize interactions between the monolayers.

An all-atom CHARMM-36 force field [40,41] was applied. The force-field parameters for DOX and CR were taken from our previous paper [42]. The TIP3P model was adopted for water [43]. The MD simulations were carried out using NAMD package [44]. Temperature and pressure were controlled by a Langevin thermostat and barostat, respectively. Van der Waals interactions were switched off at 12 Å. Electrostatic interactions were calculated with the PME method [45]. A time step of 1 fs was used in all simulations. The VMD software [46] was applied to analyze the trajectories.

Two types of MD simulations were performed. Potential of mean force (PMF) calculations [47] were done to compute the free energy profiles describing the transfer of DOX or CR across the monolayer. The *z*-coordinate of the center of mass of DOX (CR) molecule was chosen as the reaction coordinate (RC) to monitor the position of DOX (CR) with respect to the monolayer. The whole reaction path was divided into 1.5 Å long fragments. For each fragment, 0.6 ns long canonical ensemble trajectories were recorded. This allowed proper sampling of RC. Apart from biased PMF type calculations, unbiased calculations were also performed. Small-size CR or DOX/CR clusters were inserted into the monolayer and their behavior on compression was analyzed. Each simulation was composed of a 60 ns long (N,V,T) ensemble (canonical ensemble) and a 100 ns long $\left(N,\gamma ,p_{n},T\right)$ ensemble. Here, N,V,T,γ, and $p_{n}$ stand for the number of particles, volume, temperature, surface tension, and pressure normal to the surface, respectively. The equilibration was monitored by *γ* and APL values. All calculations were performed at T=293 K. The target γ was set to 40 mN∙m^−1^. The last 20 ns of the trajectories was used to compute the selected quantities.

## 4. Conclusions

Targeted delivery of drugs is of special importance especially in cases when these drugs give strong side effects. Limiting such undesired effects will affect the effectiveness of the therapy and give patients more comfort. DOX is a drug commonly used in cancer therapy. It is very effective, but, as a member of anthracycline family, it has a strong impact on healthy tissues. In this paper we have analyzed the chemotherapeutic properties of DOX/CR supramolecular complexes. It was demonstrated by Raman measurements that CR enhances DOX delivery to MCF7 breast-cancer cells. Independently, for concentration and stimulation time, CR carrier increased DOX accumulation in cancer cells. As an effect of CR presence, the uptake of DOX was about two times larger than in the reference DOX systems. CR is therefore expected to increase the permeability of DOX across biological membranes, which are natural barriers for the entry of foreign particles into the cell.

In vitro Raman studies were complemented by model monolayer experiments and molecular dynamics simulations to understand the transport phenomenon across biological membranes, a factor governing drug delivery. In line with in vitro studies, it was shown that the DOX/CR system possesses unique properties. DOX accompanied by CR exhibited enhanced penetration into the monolayer and had influence on film organization. MD simulations indicated that CR may diminish the free energy barrier for transport across the membranes. Apart from cutting DOX aggregates into smaller pieces via incorporation mechanism, CR increases adsorption to the monolayer. The adsorption in turn favors transport across the membranes. MD simulations confirmed stability of DOX/CR clusters in the monolayer and agreed qualitatively with the monolayer experiment.

Our research revealed the unique properties of the DOX/CR system, which were completely different from the reference DOX system. Taking into account affinity of immunoglobulins to bind DOX/CR supramolecular complexes, it appears that DOX/CR-containing systems can be considered as potential carriers for targeted drug transport and delivery.

## Data Availability

Results are not deposited on publicly available servers, but may be available upon request.

## Supplementary Materials

The supporting information can be downloaded at https://www.mdpi.com/article/10.3390/ijms23168935/s1.
