# Supplementary material for: Congo Red as a Supramolecular Carrier System for Doxorubicin: An Approach to Understanding the Mechanism of Action
Source: Int J Mol Sci. 2022 Aug 11;23(16):8935. doi: 10.3390/ijms23168935 (PMC9408855; doi:10.3390/ijms23168935)
Supplement: Supplementary file 1 [file ijms-23-08935-s001.zip › ijms-1861746-supplementary.pdf]

Supplementary Materials

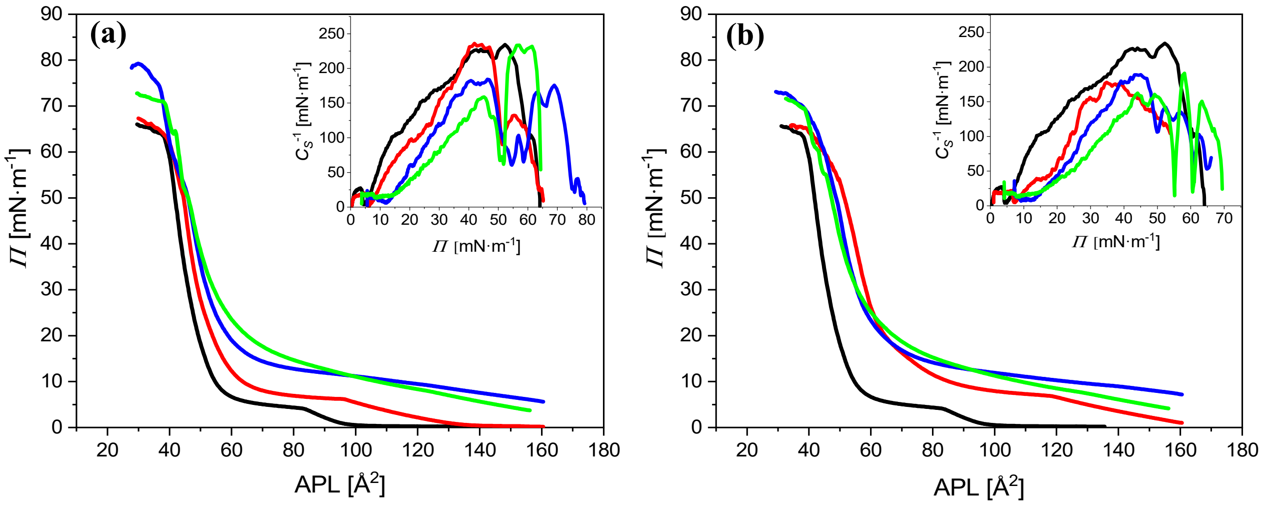

Figure S1. Compression isotherms of DPPC monolayer spread on PBS (black line), DOX (red line), CR (green line), and DOX/CR (blue line) subphase. The DOX concentration was: (a): 5 μM, and (b): 10 μM; $T = 20$ °C.

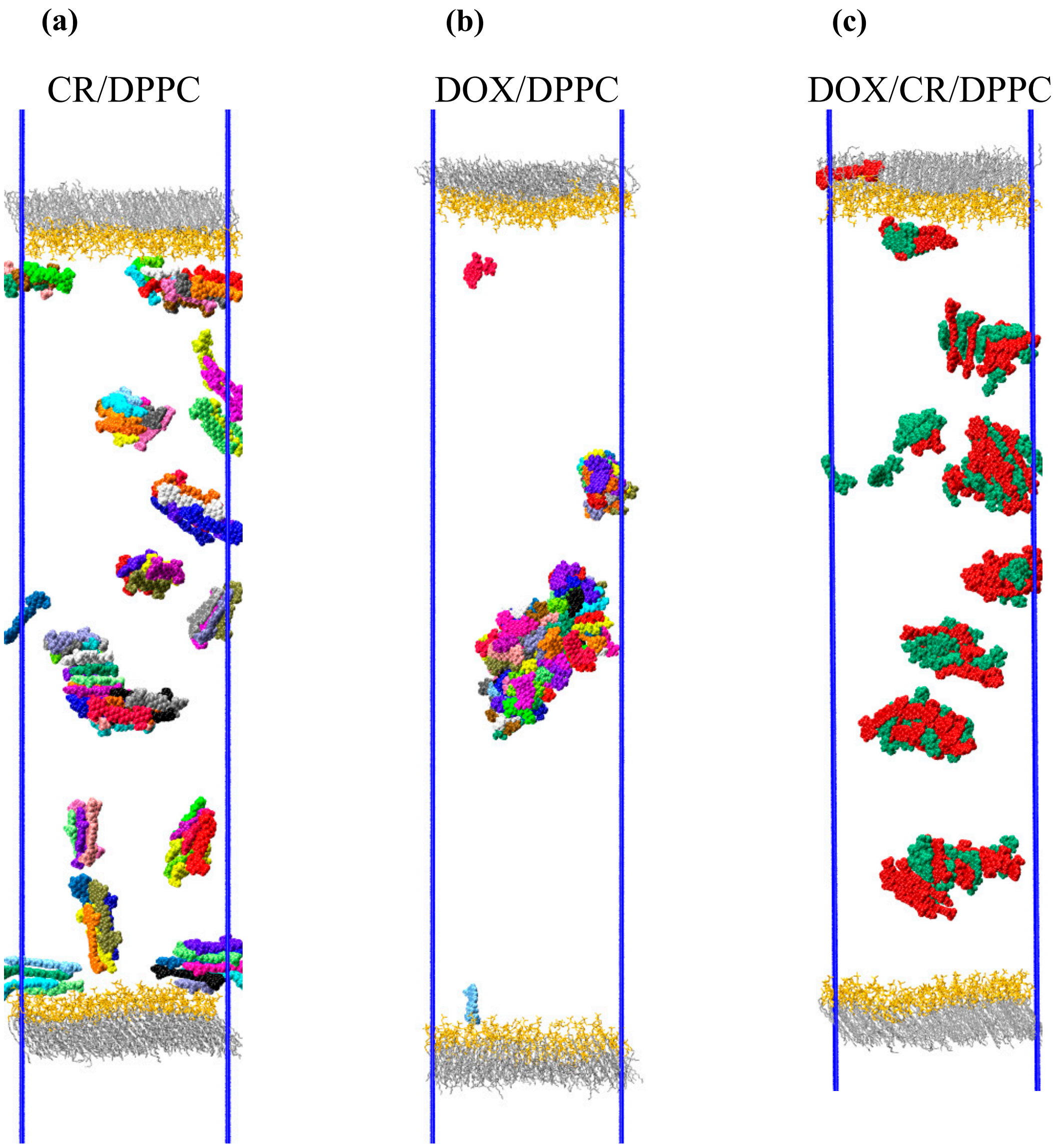

Figure S2. Side view plots of preliminary simulation boxes after 200 ns simulations for CR/DPPC (a), DOX/DPPC (b), and DOX/CR/DPPC (c). Water molecules were omitted for the clarity reason. In the CR/DPPC (DOX/DPPC) system, each CR (DOX) molecule is visualized by a different color. In a mixed DOX/CR/DPPC system, CR and DOX are red and green, respectively. The hydrophilic head groups and the hydrophobic chains of DPPC are marked in orange and silver, respectively.
